# Multilayer Hybrid Deep-Learning Method for Waste Classification and Recycling

**DOI:** 10.1155/2018/5060857

**Published:** 2018-11-01

**Authors:** Yinghao Chu, Chen Huang, Xiaodan Xie, Bohai Tan, Shyam Kamal, Xiaogang Xiong

**Affiliations:** ^1^AIATOR Co., Ltd., Block 5, Room 222, Qianwanyilu, Qianhai, Shenzhen, China; ^2^Department of Industrial and Systems Engineering, Ohio University, Athens, OH, USA; ^3^Sagacity Environment (China) Co. Ltd., A201 Qianwanyilu, Qianhai, Shenzhen, China; ^4^Indian Institute of Technology (BHU), Varanasi, Uttar Pradesh 221005, India; ^5^Harbin Institute of Technology (Shenzhen), Shenzhen 518055, China

## Abstract

This study proposes a multilayer hybrid deep-learning system (MHS) to automatically sort waste disposed of by individuals in the urban public area. This system deploys a high-resolution camera to capture waste image and sensors to detect other useful feature information. The MHS uses a CNN-based algorithm to extract image features and a multilayer perceptrons (MLP) method to consolidate image features and other feature information to classify wastes as recyclable or the others. The MHS is trained and validated against the manually labelled items, achieving overall classification accuracy higher than 90% under two different testing scenarios, which significantly outperforms a reference CNN-based method relying on image-only inputs.

## 1. Introduction

Globally, the annual solid waste is expected to reach 2.2 billion tonnes by 2025, which would cost $375.5 billion in waste management [1]. Improper waste management will have enormous adverse impacts on the economy, the public health, and the environment [1]. Municipal solid waste (MSW) recycling has been recognized as the second “most environmentally sound” strategy for dealing with urban waste by the Environmental Protection Agency (EPA) [2]. Effective waste recycling is both economic and environmentally beneficial. It can help in recovering raw resource, preserving energy, mitigating greenhouse gaseous emission, water pollution, reducing new landfills, etc [1, 3–5].

In developing country, MSW recycling relies on household separation via scavengers and collectors who trade the recyclables for profits [6–8]. In developed countries, communities are more involved in recycling program [9]. Several techniques, such as mechanical sorting and chemical sorting, are available in developed countries for automatic waste sorting [10]. However, there is huge potential to improve waste recycling even in the developed country. The municipal recycling rates of the USA and European Union are around 34% and 50%, respectively, which are significantly lower than the target recycling rate of 75% [5, 11].

The key obstacles to waste recycling include the following: (1) government plan and budget: insufficient government regulation and budget for MSW management; (2) household education: households are unaware of the importance of self-waste recycling; (3) technology: lack of effective recycling technology; and (4) management expense: the high cost of manual waste classification [1, 8, 9].

The recent progress in deep learning has contributed to unprecedented improvements in computer vision. Convolutional neural network (CNN) is one of the most recognized deep-learning algorithms for its wide application in image classification, segmentation, and detection [12–15]. Therefore in this literature, CNN is proposed to perform waste classification.

Awe et al. [16] propose an experimental project using a Faster R-CNN model to classify waste into three categories: paper, recycling, and landfill. This method achieves a mean average precision of 68%. Thung and Yang [17] deployed support vector machine (SVM) and a convolutional neural network (CNN) to classify waste into six categories. It achieves an accuracy rate of 63% for SVM and 23% for CNN. Rad et al. [18] developed a GoogLeNet-based vision application to localize and classify urban wastes. The study claims to have an accuracy rate ranging from 63% to 77% for different waste types. Donovan [19] proposed to use Google's TensorFlow and camera capturing to automatically sort waste objects as compost and recyclable. However, as a conceptual project, there is no experimental result so far. Mittal et al. [20] designed a project to detect whether an image contains garbage or not. This project employs the pretrain AlexNet model and achieves a mean accuracy of 87.69%. However, this project aims at segmenting garbage in an image without providing functions of waste classification.

As reviewed, the automatic classification methodologies available in the literature solely deploy image-based CNN and result in limited accuracy. In this work, we propose a multilayer hybrid method (MHS) to perform waste classification in public areas. Waste images associated with other numerical information measured by sensors are fed into the system. The system can automatically sort the waste item as recyclable or the others. The proposed MHS achieves a mean accuracy higher than 90%, which significantly outperforms reference image-based method.

The specific contributions of the paper are:

- Firstly, this study achieves an excellent accuracy that is useful for in-field applications: the experimental results indicate that MHS achieves an overall accuracy higher than 90%, which outperforms all reference waste classification methods in the literature.
- Secondly, this study proposes an innovative architecture to simulate the sensory and intellectual process of human inspections. While most of current waste classification methods take images as the sole input, the proposed method makes use of an AlexNet CNN to act as “human eyes” to visualize and extract key image features from its last dense layer. The system also utilizes sensors to act as “ears” and “nose” to detect other numerical feature information, which are barely discussed in the literatures. Ultimately, multilayer perceptrons (MLP) act as a response center (the “human brain”) to classify the waste object by consolidating information collected from diverse channels.

The paper is organized as follows: Section 2 introduces the inspected waste items, hardware, and data; Section 3 presents the proposed methods, including CNN, MLP, the multilayer hybrid system, and evaluation metrics; Section 4 presents the results and corresponding discussions; and conclusion are given in Section 5.

## 2. Hardware and Data

This study focuses on wastes found in urban public areas, including parks, street cleaning, landscaping, and other recreational areas. These wastes are mostly disposed of by individual visitors, pedestrians, commuters, and occasionally from commercial events. Unlike industry or household waste, the majority of municipal solid wastes are separated singular items e.g., a singular bottle or a singular lunch box [1].

This study analyzes a total of 50 different waste items that are commonly found in the investigated area [1]. Among which, 40 are recyclable and 10 are the others. The recyclable wastes are grouped into 4 main categories: paper, plastic, metal, and glass; the “others” class consists of fruit/vegetable/plant, kitchen waste, and others (Table 1). Each group is made up of representative items: the paper group, for instance, consists of books, magazines, cups, boxes, etc.  Table 2 presents the detailed information of waste items' quantity, corresponded group, and class.

### 2.1. Hardware

The proposed system consists of a high-resolution camera (model 0V9712), a bridge sensor (model HX711AD), and an inductor (model TL-W3MB1 PNP) (Table 3). The system hardware is selected based on availability, low-cost, effectiveness, and easy installation.

The camera captures images of study objects, and the images will be transferred to a PC end via USB 2.0. The bridge sensor is used to measure the weight of study object, and the inductor can detect whether the waste is made from metal or not. To our knowledge, few studies employ sensing systems for waste classification in the literature of MSW, such as applications of medical waste and wastewater sorting using weight, density, and texture detection [3]. Therefore, we propose to deploy the bridge sensor and inductor to facilitate classification of solid waste, particularly for MSW waste. Digital information measured by sensors are received and processed via an Arduino board first. The Arduino board serves as a microcontroller, which can read the outputs from the sensors, convert them into proper numerical form, and then transfer information to the PC end.

In the experiment, the investigated waste items are placed in an enclosed box with a dark grey background. The camera is placed at the upper front-right of the experiment box to maximize the marginal angle of view. Waste objects are rotated for the camera to capture views from different angles, so to simulate a three-dimension effect. The bridge sensor and the inductor are placed directly under the study object to measure their corresponding feature information.

### 2.2. Data

A total of 100 RGB images are captured for each investigated item, and 5000 (50 × 100) images in total are collected in JPG format. Each waste image is grouped with its counterpart numerical feature information as a data instance, which is then manually labelled as either recyclable or not for training/testing purpose.

**Figure 1:**
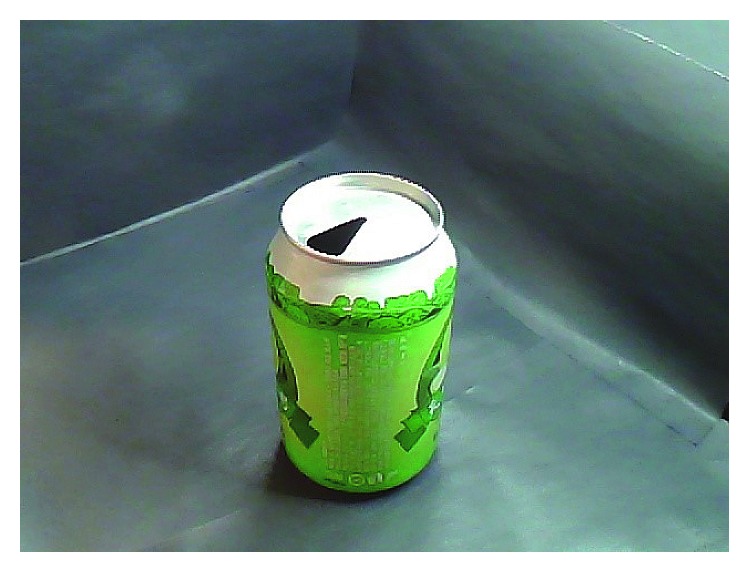
Example of the original image.

To enhance image features and to remove unwanted noise, images captured by the camera are preprocessed under the Keras framework: http://keras.io/. The original images (e.g., Figure 1) are 640 × 480 pixels in resolution while the processed images are 240 × 240. During training, 9 augmented images (Table 4), including image rotation, height/width shifting, size rescaling, zooming etc., are generated for each data instance to enhance the universality of the training model [21].

The training model has been tested twice to validate the system performance. Firstly, each waste item is placed into the system with predefined position and each item is tested for 3 times. A total of 150 (50 × 3) test set are generated for the first test. Secondly, each item is placed randomly in the system for 3 times and another set of 150 data is generated. In sum, 300 test data are created, and the system classifies each of them as recyclable or the others.

## 3. Method

A multilayer hybrid method (MHS), which consists of several subsystems, is proposed to perform waste classifications. The core of this system includes a convolutional neural network (CNN) and multilayer perceptrons (MLP). Evaluation metrics to access system performance are also discussed in this section.

### 3.1. CNN

Convolutional neural networks (CNN) are widely applied in analyzing visual image [12–15]. Generally, CNN takes images containing investigated items as inputs and classify images into different categories.

CNN is unique in its 3D volumes of a neuron: width, height, and depth. The CNN consists of a series of convolutional layers, polling layers, fully connected layers, and normalization layers [12–15]. The neurons in the convolutional layer will only connect to a small region of the previous layer. In fully connected layers, the activation neurons of the layer are fully connected to all activation neurons in the previous layer. The fully connected function can be expressed as the following forward and backward propagation rules in mathematical form:

(1) $$\begin{array}{c} X_{i}^{L+1}=\underset{i}{\sum }W_{j,i}^{L+1}X_{i}^{L},\\ g_{i}^{L}=\underset{i}{\sum }W_{j,i}^{L+1}g_{j}^{L+1}, \end{array}$$

where *X*_*i*_^*L*^ and *g*_*i*_^*L*^ represent the activation and the gradient of neurons *i* at layer *L* and *W*_*j*,*i*_^*L*+1^ is the weight connecting neurons *i* at layer *L* to neurons *j* at layer *L*+1.

The capability of CNN can be controlled by varying dimensional parameters and local architecture structure [12]. In recent years, different CNN architecture variations emerge [12, 15]. In considering the computational cost and in-field application limitations, AlexNet [12] is employed in this work.

### 3.2. AlexNet

AlexNet [12] came to the spot in the 2012 ImageNet Challenge (ILSVRC) by significantly reducing the image classification top-5 error from 26% to 15.3%. It is well-recognized for its highly capable architecture.

AlexNet contains 8 learning layers: the first five convolutional followed by three fully connected layers. The output of the last layer is fed into the 1000-way softmax which can create 1000 class labels. The kernels of the second, fourth, and fifth layers are connected to those kernels in previous layers sharing the same GPU. Kernels in the third layer, however, are fully connected to the kernels in the second layer. Response-normalization layer is associated with the first and second layers. Max-pooling layers are placed after both response-normalization layers and the fifth layer. The ReLu nonlinearity is associated with each learning layer. The neurons in the fully connected layers are connected to all neurons in the previous layer, with 4096 neurons each [12].

In this work, the network is constructed with following details:

- Layer 0: input image of size 240 × 240
- Layer 1: convolution with 96 filters, size 11 × 11, stride 4
- Layer 2: max-pooling with a size 3 × 3 filter, stride 2
- Layer 3: convolution with 256 filters, size 5 × 5, stride 1
- Layer 4: max-pooling with a size 3 × 3 filter, stride 2
- Layer 5: convolution with 384 filters, size 3 × 3, stride 1
- Layer 6: convolution with 384 filters, size 3 × 3, stride 1
- Layer 7: convolution with 256 filters, size 3 × 3, stride 1
- Layer 8: max-pooling with a size 3 × 3 filter, stride 2
- Layer 9: fully connected with 512 neurons
- Layer 10: fully connected with 512 neurons
- Layer 11: fully connected with 22 neurons

The number of the neurons in the last layers is set to 22 to equalize the number of waste categories discussed in Section 2. An additional Layer 12, which contains 1 neuron with sigmoid activation function (recyclable as 1 and others as 0), is used during training. This layer is then removed when integrated into the multilayer hybrid system that ingests the 22 outputs from Layer 11 as image features. To do so, the CNN reduces the high dimension image to low-dimensional representation with robust features only. Also, it reserves the information of important identity features which may be wrapped by final classification [22]. This application is extensively adopted in human face verification problem and face recognition as a binary classification problem.

### 3.3. MLP

Multilayer perceptrons (MLP), one of the most established deep-learning structures for nonlinear classification and regression, are frequently used for modeling and forecasting [23–26].

Neurons, which are placed in layers, are the basic processing elements of MLP. The layers between the first layer (inputs) and the last layer (outputs) are called hidden layers. The MLP employed in this work has 1 hidden layer with 10 neurons as suggested in [25] that this network is able to fit any continuous function. Neurons on each layer sum the weighted inputs, add a bias to the sum, and then apply an activation function to process the sum and compute the outputs. The signal processing of neurons can be mathematically expressed as

(2) $$\begin{array}{c} Y_{i}=\int \left(\overset{M}{\underset{j=1}{\sum }}\left(W_{ij}X_{j}+\beta_{ij}\right)\right), \end{array}$$

where *Y*_*i*_ is the output of the *i*^th^ neuron on current layer, *w*_*ij*_ and *β*_*ij*_ are the weight and bias of the *j*^th^ input on the *i*^th^ neuron, *M* is the number of inputs, *X*_*j*_ is *j*^th^ output from the previous layer, and *f* is the activation function, which is a sigmoid function in this work.

(3) $$\begin{array}{c} f\left(y\right)=\frac{1}{1+e^{-y}}. \end{array}$$

### 3.4. Multilayer Hybrid System

The multilayer hybrid system (MHS) developed in this work simulates human sensory and intelligence process system. This MHS is a combination of several interdependent subsystems, including: (1) image system (2) sensor system and (3) the central back-end classification system.

**Figure 2:**
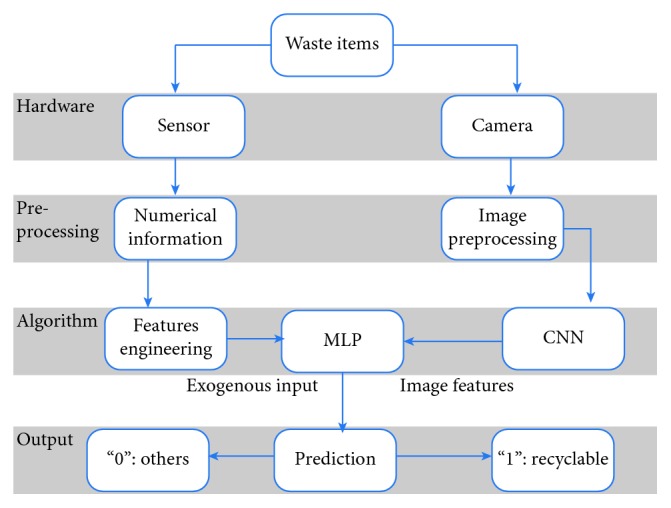
Multilayer hybrid system (MHS).

Figure 2 illustrates the multilayer hybrid system with three interacting subsystems and their associated components. The arrows indicate the processing flow and interaction of subsystems.

When a waste item is received into the hybrid system, the camera and sensors are activated to observe the item. The imaging system consists of a camera to capture an image, which is analyzed by the CNN. The sensor system, simultaneously, functions to obtain numerical information from the objects. The ultimate results (binary output) are obtained using MLP system, whose inputs are the 22 outputs from the CNN and the numerical information from sensors. In this respect, MLP system can be trained independently from CNN model, and the weight and bias parameters of CNN model can be kept unaffected. On the other hand, as the outputs of CNN are the inputs for MLP, these two models actually function simultaneously to generate the binary classification results.

### 3.5. Evaluation Metrics

Each classification prediction by the proposed system is compared to the manually classified label, which is set as the “truth”. The confusion matrix shown in Table 5 quantifies the hits and misses of the automatic classification system. A CNN-only model with exactly the same structure discussed above is trained and evaluated as a reference model.

The system performance is evaluated with accuracy, precision, and recall. The accuracy of classification is defined as the percentage of images that are correctly classified:

(4) $$\begin{array}{c} \text{accuracy}=\frac{\left(\text{TP}+\text{TN}\right)}{\left(\text{TP}+\text{TN}+\text{FP}+\text{FN}\right)}\times 100\text{\%}. \end{array}$$

Precision, therefore, represents the correctness of classification prediction systems.

(5) $$\begin{array}{c} \text{Precision}=\frac{\text{TP}}{\left(\text{TP}+\text{FP}\right)}\times 100\text{\%}. \end{array}$$

Recall represents the effectiveness of classification prediction systems.

(6) $$\begin{array}{c} \text{Recall}=\frac{\text{TP}}{\left(\text{TP}+\text{FN}\right)}\times 100\text{\%}. \end{array}$$

Incorporating precision and recall can reduce the bias forecasting caused by the unbalanced dataset: the minority class is harder to learn and the model tends to over forecast the majority class with highly skew data [26].

## 4. Result and Discussion

The MHS model, which is trained using 5000 data instances, is evaluated under two different scenarios: the item is placed with fixed and random orientations. The model performances are compared with a CNN model that takes the images as the only input. The classification results from each model are presented in Table 6.

The evaluation results presented in Table 6 indicate that MHS significantly outperforms CNN-only model in terms of all three matrices (accuracy, precision, and recall), particularly for the “others” category.

MHS achieves accuracy rates above 90% for both the first and second tests, which are 10% higher than the CNN-only model (Table 6). The MHS model also achieves higher precision rate of 98.5%, 97.1% to 88.6%, 85.9%, respectively, indicating MHS model's effectiveness in predicting recyclable items. In addition, the MHS model shows great performance in recall (99% and 92%, respectively, for the first and second tests), showing that MHS is highly sensitive in identifying dedicated recyclable waste items.

The following tables display three sets of representative items returning from testing results.  Table 7 represents items that are correctly classified by both MHS and CNN; Table 8 represents items that are correctly classified by the MHS but incorrectly classified by the CNN.  Table 9 consists of items with low classification accuracy in both models.

It can be noticed that both MHS and CNN perform well when investigated items have strong image features (Table 7). However, CNN performs poorly when waste items lack distinctive image features, especially for “other” waste.

For instance, the images of beer cap and the transparent box (Table 8) are weak to be distinguished from the experiment background. It is difficult for the CNN to extract their imagery features in training, thus fail in testing. The cabbage item is irregular in appearance showing different figures for different orientations of placement. The CNN model itself is not sufficient enough to construct the feature patterns for accurate classification. The egg's figure, on the other hand, is too simple to transfer sufficient information for training model resulting in a limited performance.

CNN relies on image information only, and if the study items are weak in imagery features, its classification performance will be adversely affected. MHS can address the problem by integrating both image and other feature information. In situations where the image information is insufficient, MHS is able to take advantages of other useful feature to make the most appropriate classification decision. Nevertheless, for items whose image features and other numerical features are weak, their MHS classification error may increase. For instance, the MHS accuracy rate of the cup is about 60% (Table 9). After inspecting each individual misclassification case, we found that the major reason to cause these errors is that these objects have cylinder shapes, which are usually misclassified as bottles that are recyclable.

## 5. Conclusion

An automatic classification system based on multilayer hybrid deep learning (MHS) is proposed to classify disposal of waste in the urban public area. The system simulates human sensory and intelligence process system by deploying a high-resolution camera together with multiple functional sensors. The multilayer hybrid method consists of three interdependent subsystems, including an image processing system, a numerical sensor system, and a multilayer perceptrons (MLP) system. The image processing system deploys AlexNet CNN to extract waste imagery information as inputs for the MLP. The sensor system aims at measuring other waste features as numerical input for MLP. The MHS is used to automatically classify the waste item as either recyclable or the others by consolidating information from both image and sensory channels.

A total of 50 waste items are used to evaluate the performance of MHS, which is also compared with a CNN-only model that only takes images as input. The result indicates that the MHS achieves a significantly higher classification performance: the overall performance accuracies are 98.2% and 91.6%, (the accuracy of the reference model is 87.7% and 80.0%) under two different testing scenarios.

This study demonstrates the potential of the proposed MHS in improving waste classification's efficiency and effectiveness. In considering the continually increased volume of waste globally and the urgent requirements for environmentally friendly waste processing, the proposed MHS is both economically and environmentally beneficial.

## Figures and Tables

**Table 1:** Representative waste images.

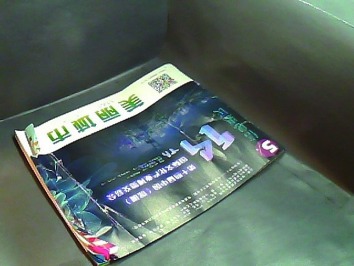	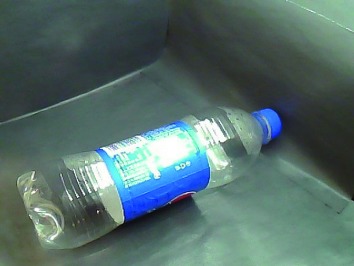
Paper	Plastic
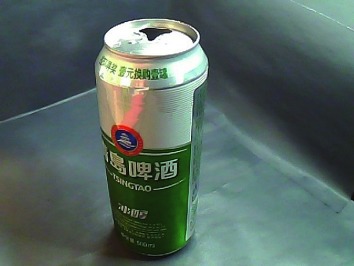	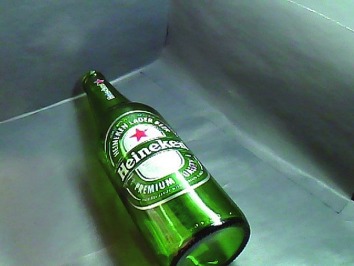
Metal	Glass
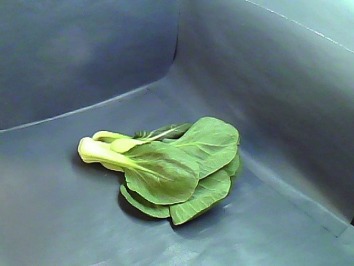	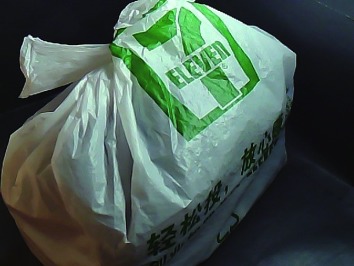
Fruit/vegetable/plant	Others

**Table 2:** Waste item.

Class	Group	Item	Quantity
Recyclable	Paper	Books	5
		Cups	5
		Boxes	4
	Plastic	General bottles	6
		Shampoo bottles	4
		Pen	1
		Watch	1
	Metal	Cans	7
		Key	1
		Scissor	1
		Beer cap	1
	Glass	Bottle	4
*Sum*	**4**	**12**	**40**

Others	Fruit/vegetable/plant	Apple	1
		Banana	1
		Carrot	1
		Cabbage	1
		Rose	1
		Others	1
	Kitchen waste	Egg	1
		Lunch box	1
	Others	Trash bag	1
		Bowl	1
*Sum*	**3**	**10**	**10**

**Table 3:** Experiment sensors.

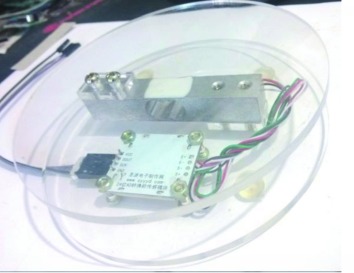	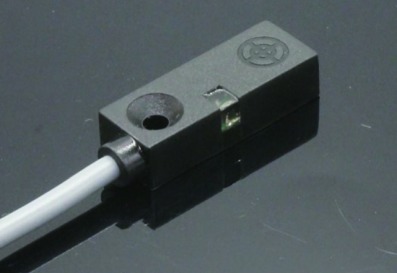
Bridge sensor	Inductor

**Table 4:** Example of images generated by the augmentation algorithm.

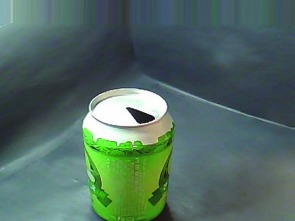	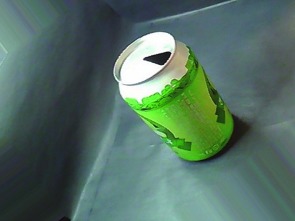	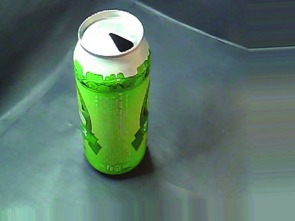
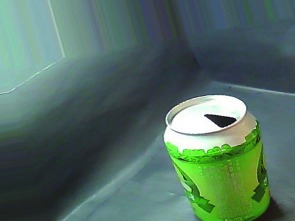	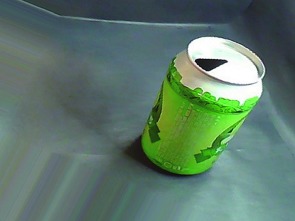	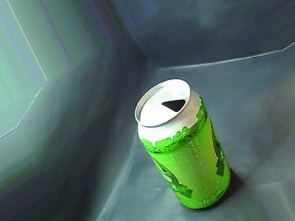
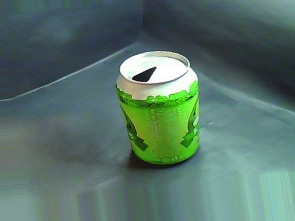	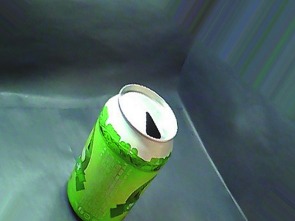	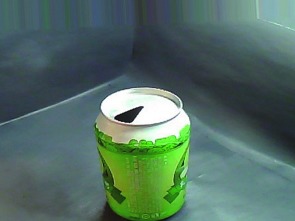

**Table 5:** Confusion matrix of automatic waste classification for each category.

Automatic classification	Manual classification	
	Recyclable	Others
Recyclable	True positive (TP)	False positive (FP)
Others	False negative (FN)	True negative (TN)

**Table 6:** Confusion matrices for different classification models.

Evaluation metrics	MHS model		CNN model	
	1^st^ test	2^nd^ test	1^st^ test	2^nd^ test
Accuracy (%)	98.2	91.6	87.7	80.0
Precision (%)	98.5	97.1	88.6	85.9
Recall (%)	99.3	92.3	96.8	89.2

**Table 7:** Representative waste items that are correctly classified by both MHS and CNN.

MHS	CNN		
✓	✓	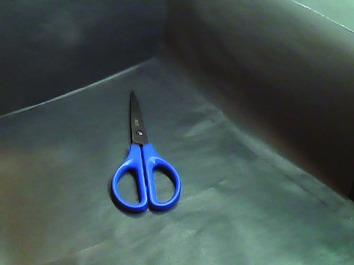	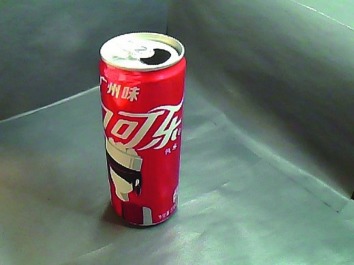
		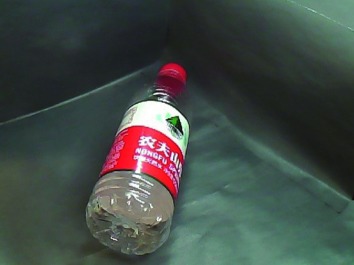	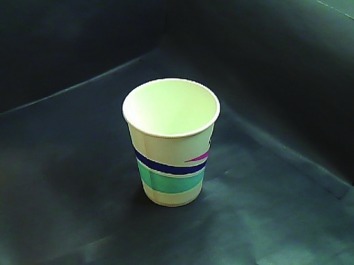

**Table 8:** Representative waste item that are correctly classified by the MHS but incorrectly classified by the CNN.

MHS	CNN		
✓	X	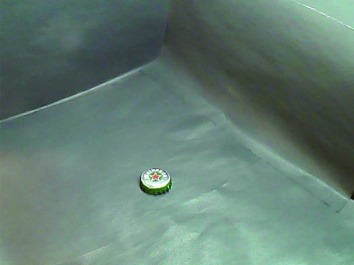	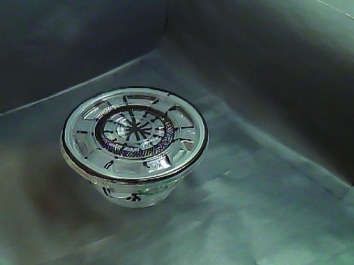
		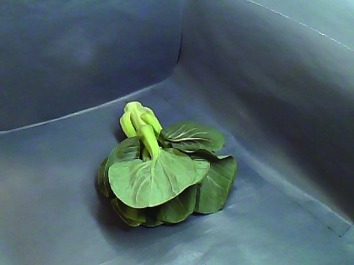	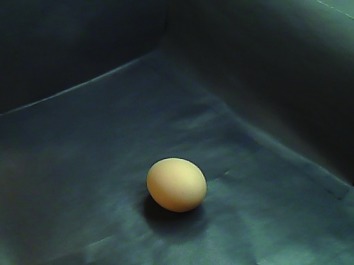

**Table 9:** Representative waste item with low accuracy in MHS and CNN.

MHS	CNN		
X	X	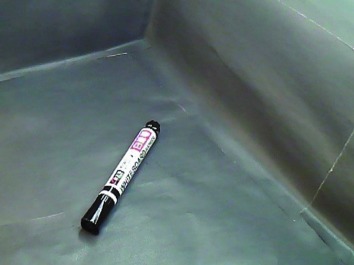	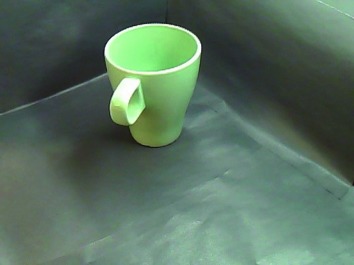

## Data Availability

The image and numerical data used to support the findings of this study are available from the corresponding author upon request.
